# Characterization of the Cytopathic Effects of Monkeypox Virus Isolated from Clinical Specimens and Differentiation from Common Viral Exanthems

**DOI:** 10.1128/jcm.01336-22

**Published:** 2022-11-29

**Authors:** Angela Ma, Janine Langer, Kimberly E. Hanson, Benjamin T. Bradley

**Affiliations:** a Department of Pathology, University of Utah, Salt Lake City, Utah, USA; b ARUP Laboratories, Salt Lake City, Utah, USA; c Division of Infectious Diseases, Department of Medicine, University of Utah, Salt Lake City, Utah, USA; Cepheid

**Keywords:** monkeypox virus, MPXV, viral culture, cytopathic effects, viral load, virology, culture, virus

## Abstract

While the practice of viral culture has largely been replaced by nucleic acid amplification tests, circumstances still exist in which the availability of viral culture will allow for the diagnosis of infections not included in a provider’s differential diagnosis. Here, we examine the cytopathic effects (CPE) and clinical data associated with 18 cases of monkeypox virus (MPXV) isolated from 19 clinical samples submitted for viral culture. During the study period, a total of 3,468 viral cultures were performed with herpes simplex virus (HSV) most commonly isolated (646/3,468; 18.6%), followed by MPXV (19/3,468; 0.6%) and varicella-zoster virus (VZV) (12/3,468; 0.4%). Most MPXV-positive samples were obtained from males (14/19) and taken from genital (7/19) or rectal lesions (5/19). Cycle threshold values of tested samples ranged from 15.3 to 29.0. Growth of MPXV in cell culture was rapid, yielding detectable CPE at a median of 2 days (range: 1 to 4) often with >50% of the monolayer affected in RMK, BGM, A549, and MRC-5 cell lines. As clinical features of MPXV, HSV, and VZV lesions may overlap, CPE patterns were compared between viruses. HSV CPE developed in a similar time frame (median: 2 days, range: 1 to 7) but was more often negative in RMK cells relative to MPXV. VZV grew more slowly (median: 9 days, range: 5 to 11) and demonstrated CPE affecting ≤25% of cell monolayers when positive. Viral culture remains an important tool for the detection of rare or emerging viral pathogens, particularly when high viral load specimens are easily obtained.

## INTRODUCTION

The 2022 monkeypox virus outbreak has rapidly evolved since the first confirmed case in a United Kingdom traveler returning from Nigeria on May 7th, 2022 (1, 2). Following evidence of sustained transmission in non-endemic countries, the outbreak was declared a Public Health Emergency of International Concern by the World Health Organization and US Department of Health and Human Services (3, 4). Monkeypox virus (MPXV) is a member of the *Orthopoxivirus* genus, which is comprised of other human pathogens including cowpox, vaccinia, and variola viruses, the latter being the causative agent of smallpox disease (5). Human infection with MPXV was first recorded in 1970, and although the zoonotic reservoir remains unknown, rodents are suspected to be the most likely source (6). Two phylogenetic clades of MPXV are recognized, Congo Basin (Central African) and West African (7). Infection with the Congo Basin lineage is associated with more severe illness resembling smallpox and is classified as a select agent by the US Centers for Disease Control and Prevention (CDC), whereas West African monkeypox infection typically demonstrates decreased clinical severity and transmission (8). The current monkeypox outbreak has been attributed to the West African clade (1). Predominantly endemic to regions of Africa, sporadic cases of travel-associated monkeypox have been documented in other countries without sustained human-to-human transmission. Prior to the current outbreak, the last US monkeypox outbreak was reported in 2003 and was associated with infected prairie dogs exposed to rodents originating from Ghana (9). Human-to-human transmission of monkeypox was presumed to infrequently occur; however, direct contact between individuals has been a predominant driver in the spread of the 2022 outbreak (4). Preliminary data suggests decreasing population immunity due to termination of smallpox vaccine administration and novel mutations in outbreak-associated isolates have contributed toward increased human transmission (10).

Nucleic acid amplification tests (NAATs) have largely become the primary diagnostic method of viral infections due to their rapid turnaround times and increased sensitivity relative to the historical gold standard, viral culture. While conventional viral culture is more laborious and resource intensive, its utility during novel pathogen outbreaks should not be dismissed (11). Viral culture can detect a wide range of pathogens not covered by common NAATs and isolate viruses that may not have been part of a provider’s original differential diagnosis (12). Furthermore, culture growth is less impacted by strain variability or genetic mutations that may contribute to false negative results by NAATs (13). Viral culture is necessary for phenotypic antiviral susceptibility testing and culture results can be useful in determining infectivity in situations of prolonged viral shedding (14, 15). Few studies have documented viral culture of MPXV with most being limited to the Congo Basin lineage (16–18). Moreover, the appearance of cytopathic effects (CPE) caused by MPXV has been poorly characterized from the standpoint of the practicing clinical microbiologist.

At Associated Regional and University Pathologists, Inc. (ARUP Laboratories), viral cultures are routinely performed on respiratory and non-respiratory sources using shell vials. Through this technique, ARUP Laboratories identified its first case of monkeypox infection from the current outbreak on July 11^th^, 2022, 2 weeks prior to the availability of an in-house orthopoxvirus molecular assay. In this study, we examined the growth characteristics and CPE of MPXV isolated from primary clinical specimens. MPXV CPE were also compared to herpes simplex virus (HSV) and varicella-zoster virus (VZV), which may cause similar lesions.

## MATERIALS AND METHODS

### Data collection.

Laboratory records were reviewed to identify viral culture test results from July 11^th^, 2022 to August 26^th^, 2022. Demographic information and specimen handling timestamps were recorded. For specimens with unusual CPE that were ultimately confirmed to be MPXV positive by molecular testing, the laboratory information system was queried to see if any additional microbiological testing had been ordered in the 48 h surrounding specimen collection. For comparison of HSV and VZV CPE to MPXV CPE, 20 HSV-positive samples and 11 VZV-positive samples were evaluated. An IRB exemption was granted by the University of Utah IRB under ID#: 00158025.

### Viral culture.

Swabs were shipped in viral transport media at approximately 4°C and received at ARUP Laboratories. Upon receipt, specimens were processed for viral culture. Briefly, the tube was vortexed to release bound cells or virus, and transport media was aliquoted into a labeled holding tube where a 0.2 mL mixture of penicillin, streptomycin, and Amphotericin B was added. The tube was then centrifuged at 1500 × *g* for 10 min to clarify the sample. Shell vials containing cell lines were each inoculated with 0.2 to 0.3 mL of the treated supernatant. Depending on the test ordered, individual shell vials containing rhesus monkey kidney cells (RMK) (Quidel), Buffalo green monkey kidney cells (BGM) (ARUP Reagent Laboratory), A549 human lung carcinoma (ARUP Reagent Laboratory), or MRC-5 human embryonic lung fibroblast cell lines (ARUP Reagent Laboratory) were inoculated. Cell lines used for each assay were as follows: Non-respiratory broad viral culture with or without CMV DFA: RMK, BGM, A549, and MRC-5 cell lines; Herpes simplex viral culture: A549 cell line; Varicella-zoster viral culture: A549 and MRC-5 cell lines. Inoculated cell lines were incubated at 37°C, 5% CO_2_ in 1 mL of Minimum Essential Maintenance Media (ARUP Reagent Laboratory) with 2% fetal bovine serum (Avantor Seradigm) and 50 U/mL penicillin, 30uM streptomycin, 13.8 uM vancomycin, 1.08 uM Amphotericin B, and 0.02 mg/mL gentamicin. Cultures were observed daily over a total of 14 days for the development of CPE. In this study, CPE are defined as morphologic changes to the unstained cell monolayer observable by light microscopy. Changes may include cell discohesion (either globally or in foci with a central clearing), cell rounding, or syncytia formation. When present, CPE were graded as follows: 0 = no CPE present, 1+ = ≤25% CPE, 2+ = >25% CPE, 3+ = >50% CPE, 4+ = >75% CPE. CPE were graded for all cell lines on the first day in which any cell line demonstrated CPE. Images of CPE were acquired using an inverted light microscope.

For cultures with typical CPE, diagnosis was confirmed by direct immunofluorescent (DFA) or indirect immunofluorescent (IFA) staining of the shell vial. DFA kits were used to test for herpes simplex virus types 1 and 2 (D^3^ DFA HSV Identification and Typing kit, Quidel), varicella-zoster virus (Light Diagnostics, Varicella-Zoster Virus DFA kit, EMD Millipore), adenovirus (D^3^ Ultra Respiratory Virus Screening & Identification kit, Quidel), and cytomegalovirus (D^3^ DFA Cytomegalovirus Immediate Early Antigen Identification kit, Quidel). IFA kits were used to test for enterovirus (D^3^ IFA Enterovirus Identification and Typing kit, Quidel). Manufacturer instructions were followed for all kits.

Workup of cultures with atypical CPE changed over the course of the study. Initially, these specimens were tested by DFA and IFA reagents to common viruses including HSV, VZV, adenovirus, and enterovirus. If workup was negative for these viruses, a presumptive diagnosis of MPXV was made. The medical directors then contacted the client to discuss ancillary molecular testing through state and local public health labs. Once laboratory staff became more familiar with the prototypical CPE of MPXV and following the availability of on-site molecular testing, cultures demonstrating CPE consistent with MPXV had their work-up stopped and were sent to orthopoxvirus NAAT for confirmation.

### ARUP *Orthopoxvirus* real-time PCR assay.

Presumptive detection of MPXV from clinical samples was performed by a laboratory-developed real-time PCR assay using primers and probes designed by ELITech (Bothell) targeting a conserved region within the polymerase gene common among the *Orthopoxvirus* genus. Samples received on dry swabs were submerged in 500 μL of PBS, vortexed for 15 s, then allowed to sit at room temperature for 1 h before proceeding to extraction. For specimens in VTM and dry swabs following resuspension, 200 μL of sample was eluted into 80 μL on the Chemagic MSMI (PerkinElmer) instrument. Following reaction set-up, amplification was performed on the QuantStudio 12K Flex (ThermoFisher) to determine the cycle threshold.

Prism (version 9.4.1) was used for graphical and statistical analysis. An unpaired t test was performed to compare the Ct values between positive specimens sent for viral culture to those sent for orthopoxvirus NAAT only. A Kruskal-Wallis test with *post hoc* analysis was conducted to examine for differences in CPE intensity between different cell lines for each virus.

## RESULTS

### Epidemiological and laboratory data for positive monkeypox virus specimens.

Over the course of the study, a total of 1,482 non-respiratory broad viral cultures, 1,674 herpes simplex viral cultures, 173 varicella-zoster viral cultures, and 139 non-respiratory broad viral cultures with CMV DFA were performed. The highest positivity rate of MPXV detection was from the non-respiratory broad viral culture (14/1482; 0.9%), followed by non-respiratory broad viral culture with CMV DFA (1/139, 0.7%), varicella-zoster viral culture (1/173, 0.6%), then herpes simplex viral culture (3/1674, 0.2%). For the non-respiratory broad viral culture assay, MPXV was detected more frequently (*n* = 14) than all other viruses (VZV, *n* = 11; Enterovirus, *n* = 3; Adenovirus, *n* = 2) excluding HSV (*n* = 170) (Table 1).

**TABLE 1:** Positivity rates of non-respiratory viral culture tests performed during study period

Test	Positive cultures (%)	Proportion of positive cultures (%)
Non-respiratory viral culture (*n* = 1482)	200 (13.4)	
HSV	170 (11.5)	85
Monkeypox	14 (0.9)	7
VZV	11 (0.7)	5.5
Enterovirus	3 (0.2)	1.5
Adenovirus	2 (0.1)	1
HSV culture (*n* = 1674)	447 (26.7)	
HSV	444 (26.5)	99.3
Monkeypox	3 (0.2)	0.7
VZV culture (*n* = 173)	32 (18.5)	
HSV	30 (17.3)	93.8
VZV	1 (0.6)	3.1
Monkeypox	1 (0.6)	3.1
Non-respiratory viral culture with CMV DFA (*n* = 139)	3 (2.1)	
HSV	2 (1.4)	67
Monkeypox	1 (0.7)	33

The 19 positive MPXV samples originated from 18 individuals including 14 males and 4 females. The median age for individuals with cell culture results suggestive of MPXV infection was 33 (range: 9 to 51 years). The predominate specimen sources were genital (7/19) and rectal (5/19). Less frequent specimen sources included skin (2/19), upper extremity (2/19), foot (1/19), pharynx (1/19), and 1 specimen for which a source was not provided. For many individuals (9/18), no additional infectious disease tests were requested from our lab within 48 h of viral culture specimen collection. When additional orders were placed, they included assays to detect HSV (*n* = 8), orthopoxvirus (*n* = 4), and VZV (*n* = 2). On rare occasions, testing was performed for tick-borne diseases (Table 2).

**TABLE 2:** Descriptive characteristics of monkeypox virus isolated from viral culture (*n* = 19)

Median age in yrs (range)	33 (9 to 15)
Gender (n [%])	
Male	14 (73.7)
Female	4 (26.3)
Median turnaround time in hours (range)	
Collection to receipt	40.2 (5.2 to 75.4)
Receipt to result	87.4 (29.3 to 214.6)
Overall time	127.6 (53.5 to 256.0)
Day of incubation CPE first detected (n [%])	
Day 1	1 (5.3)
Day 2	9 (47.4)
Day 3	5 (26.3)
Day 4	4 (21.0)
Specimen source (n [%])	
Genital (including vagina, groin)	7 (36.8)
Rectal (including anal)	5 (26.3)
Skin	2 (10.5)
Upper extremity (including hands, fingers)	2 (10.5)
Foot	1 (5.3)
Pharynx	1 (5.3)
Not specified	1 (5.3)
Additional infectious disease tests ordered (n)	
No additional testing	9
Orthopoxvirus NAAT	4
HSV (including culture, DFA^*a*^, NAAT)	8
VZV (including DFA^*a*^, NAAT)	2
HIV (including 1/2 differentiation, NAAT)	2
AFB tissue culture	2
*Ehrlichia* and *Anaplasma* NAAT	1
Rickettsia rickettsii serology	1
Acute hepatitis serology	1
Orthopoxvirus NAAT testing	
Cases confirmed (n [%])	19 (100)
Avg Ct value^*b*^ (range)	20.8 (15.3 to 29.0)

a Direct fluorescent antibody stain for HSV-1/2 and VZV performed on primary specimen.

b In-house testing performed on 18/19 specimens.

### Monkeypox virus is rapidly detected from viral culture.

**FIG 1:**
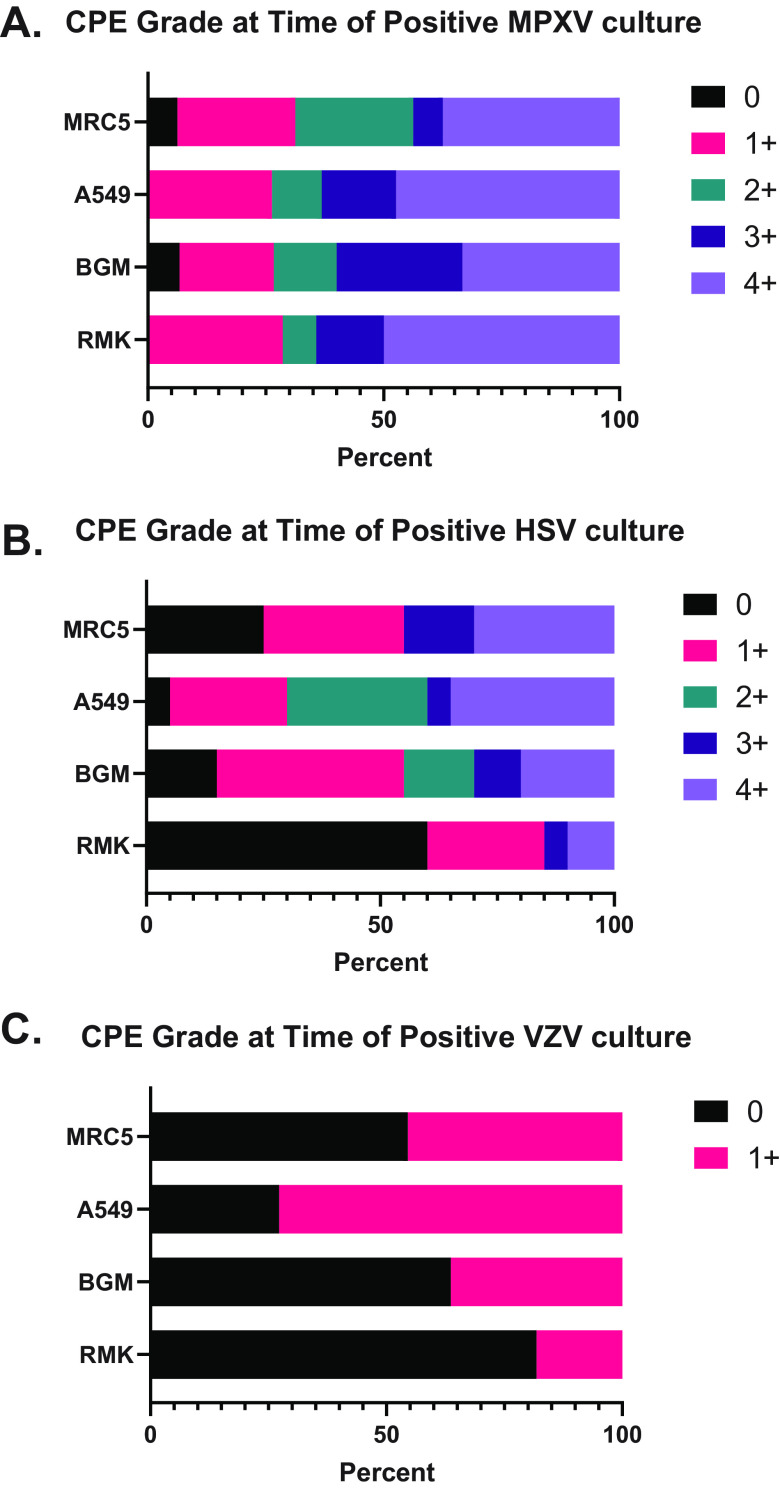
CPE grade at the time of positive culture for MPXV (A), HSV (B), and VZV (C). CPE graded as follows: 0 = no CPE present, 1+ = ≤25% CPE, 2+ = >25% CPE, 3+ = >50% CPE, 4+ = >75% CPE.

Cytopathic effects consistent with MPXV was rapidly detected from cell culture. Overall, CPE were demonstrated at a median of 2 days following inoculation. Of the 19 samples, 1 was positive at Day 1, 9 at Day 2, 5 at Day 3, and 4 at Day 4 (Table 2 and Table S1). Each of the 4 cell lines used in our assays appeared permissible to MPXV replication. Only 1 specimen (MPXV_0012) did not demonstrate CPE in all cell lines on the first day of positivity (Table S1). For this specimen, both the MRC-5 and BGM cell lines were negative but turned positive 2 days later. The degree of CPE varied between different cell lines with half (7/14) of the RMK cells demonstrating a CPE of 4+ while only 35% (5/14) of BGM cells had a CPE of 4+ on the first positive day (Fig. 1A). Differences in CPE grade between cell lines did not achieve statistically significant levels (Kruskal-Wallis test, *P* > 0.05).

**FIG 2:**
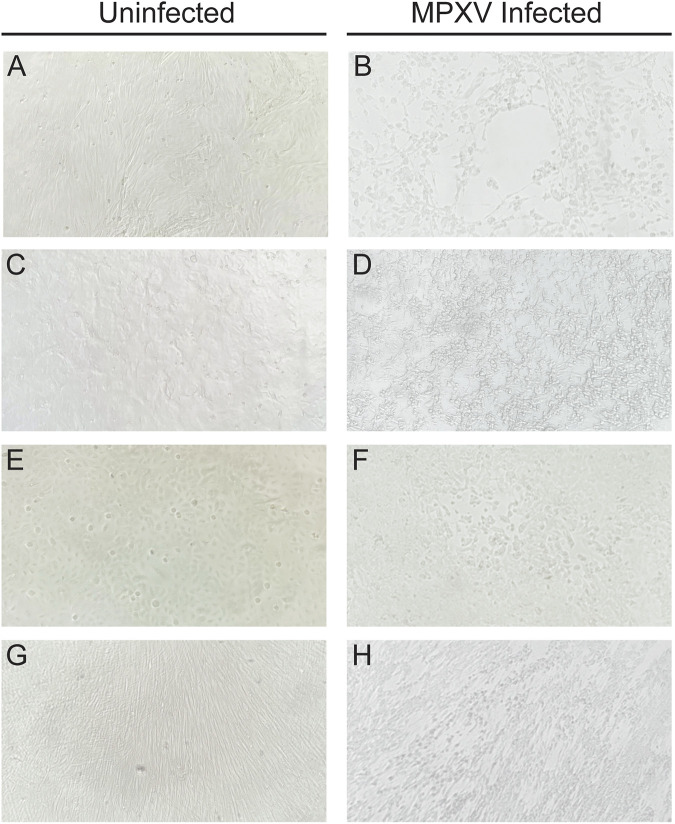
Comparison of uninfected cells with the cytopathic effects of MPXV following 2 days incubation at 37°C, 5% CO_2_ in RMK (A) and (B), BGM (C) and (D), A549 (E) and (F), and MRC-5 (G) and (H) cell lines. Images taken at ×100 magnification.

Infection with MPXV led to significant, observable changes to cell culture monolayers. Uninoculated RMK cells demonstrate a biphasic population of fibroblasts and epithelial cells. When MPXV CPE were present, there was a significant degree of cellular detachment and degeneration. The remaining cells were refractile and demonstrated pronounced cytoplasmic bridging. Given the extent of CPE, preferential infection of fibroblasts or epithelial cells were unable to be determined by light microscopy. Uninoculated heteroploid BGM cells typically demonstrate a flat, cuboidal morphology, and when infected with MPXV these cells demonstrated clumping with pinching of the cytoplasmic membrane. In the A549 cells which are typically described as “cobblestone” in appearance, MPXV CPE manifested as foci of cellular detachment with surrounding cells demonstrating a glassy or “melted” appearance of their membranes. The diploid fibroblast cell line, MRC-5, grows as sheets of streaming, spindled cells in a woven appearance. MPXV CPE in these cells demonstrated cell degeneration with chords of rounded fibroblasts alternating with areas of preserved cell growth. The low-magnification appearance of streaming CPE tracks alternating with uninvolved cells was described as a “hurricane” pattern by the lab to evoke the image of the radial bands of hurricane clouds when viewed from above (Fig. 2).

### Growth characteristics and CPE patterns allow for separation of MPXV from HSV and VZV.

As HSV and VZV are normally the predominate viruses isolated by culture from non-respiratory sources, the CPE of these viruses were compared to that of MPXV. A random subset of 20 HSV cases and all 11 VZV cases isolated from the non-respiratory broad viral culture assay were used for analysis. In comparison to MPXV, HSV demonstrated a similar median time to positivity of 2 days (range 1 to 7 days), however, a higher majority of HSV cases were positive on Day 1 in comparison to MPXV. When observable CPE were detected from HSV culture, it was most frequently seen in A549 cells (19/20, 95%) followed by BGM cells (17/20, 85%). Differences in CPE between cell lines only reached statistical significance for A549 versus RMK cells (Kruskal-Wallis test; *P* < 0.05). The CPE grade was lower in comparison to MPXV in all cell lines. Notably, over half of RMK cells were graded as 0 for HSV while 50% (7/14) were graded as 4+ for MPXV (Fig. 1A and B). VZV cultures were positive at a significantly later time, giving positive results at a median of 9 days (range 5 to 11 days) (Table S1). VZV isolates demonstrated less CPE in comparison to MPXV and HSV (Fig. 1C). CPE were most commonly observed in A549 cells (8/11, 73%). On the initial positive CPE read for all VZV isolates, no specimen had a CPE grade greater than 1+. As with HSV, the RMK cell line demonstrated the least amount of CPE (2/11, 18%); however, differences in CPE intensity between cell lines did not reach statistically significant levels.

**FIG 3:**
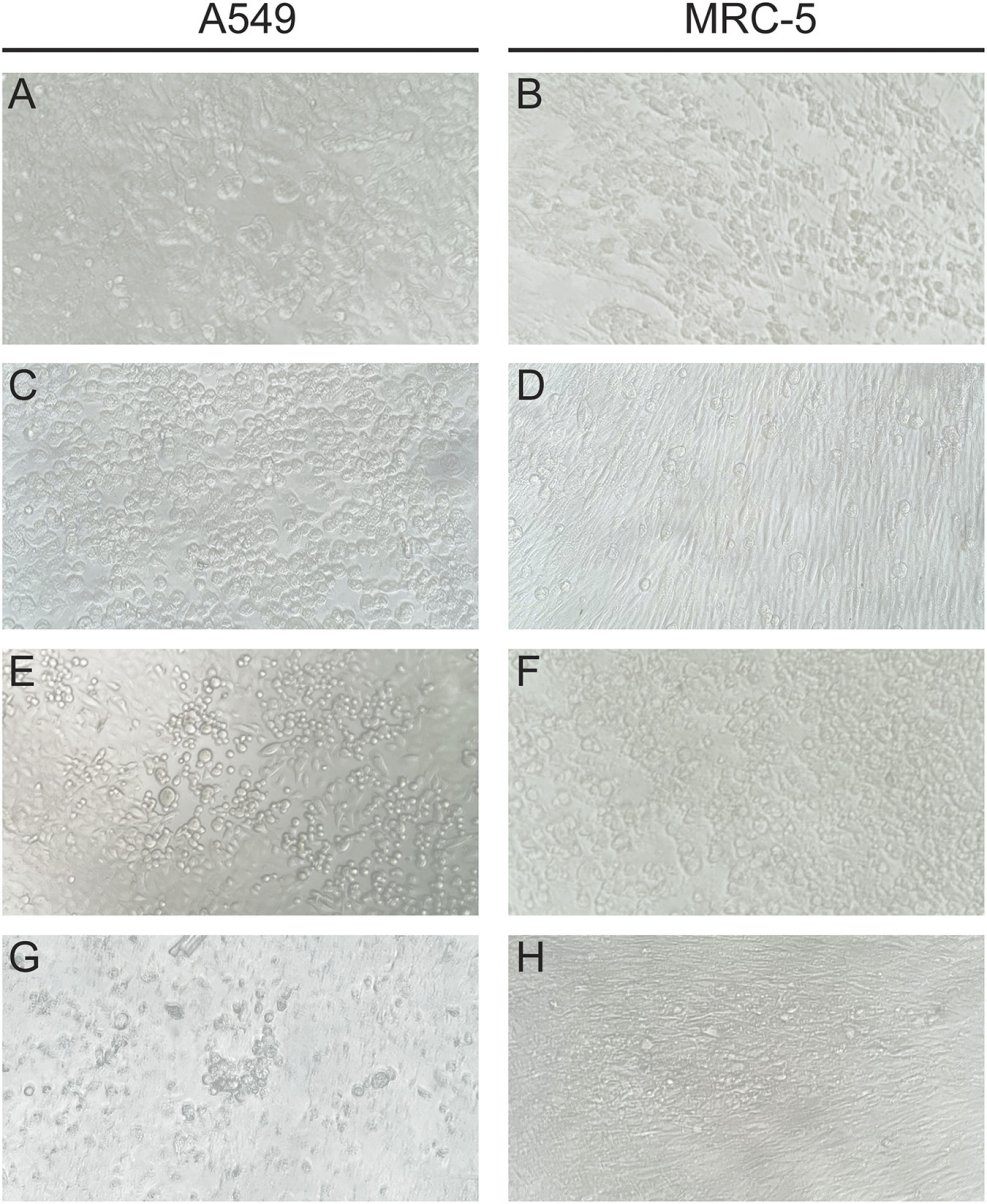
Comparison of typical MPXV (A) and (B), HSV1 (C) (D), HSV2 (E) and (F), and VZV (G) and (H) cytopathic effects in A549 and MRC-5 cells. Images taken at ×200 magnification.

In addition to growth characteristics, observable differences in the CPE of MPXV, HSV, and VZV exist. The A549 cell line potentially provides the greatest resolution as different features are observed for HSV1, HSV2, VZV, and MPXV. HSV1 and HSV2 typically present as rounded, ballooned cells with HSV2 demonstrating a wider variation in cell size (Fig. 3C and E). VZV CPE in A549 cells present as foci of less refractile, rounded cells sometimes surrounding a central clearing (Fig. 3G). MPXV CPE of A549 cells also show a central clearing though the cells have less distinct borders and a more refractile appearance (Fig. 3A). For the MRC-5 cell line, the CPE pattern is similar between HSV and VZV where it presents as single or small clusters of rounded cells and contrasts to the broader patches formed by MPXV (Fig. 3B, D, F, and H). Within BGM cells, HSV CPE manifest as large, web-like foci of bridging cells while VZV CPE occur in tighter foci (Fig. S1C, E, and G). Similar to HSV, the CPE of MPXV occur in patches though the foci may be more elongated (Fig. S1A). Extensive CPE (>75% cell monolayer affected) are infrequently observed in RMK cells infected with HSV, and in this study, 10% (2/20) were observed at 4+. When present, HSV CPE in RMK cells demonstrate scattered rounded cells with refractile bodies similar to what is seen in the A549 cell line (Fig. S1D and F). VZV CPE in RMK are present with a central clearing surrounded by cells with cytoplasmic bridging (Fig. S1H). The CPE of MPXV in RMK cells are much more pronounced with a significantly higher rate of 4+ CPE. These cells demonstrate conspicuous cytoplasmic bridging and rapid monolayer degeneration with cells floating in the media (Fig. S1B). Descriptive findings for CPE in each virus and cell line combination are summarized in Table 3.

**TABLE 3:** Comparison of MPXV CPE to viruses commonly isolated from non-respiratory sites

Virus	Avg days to produce CPE	Permissible cell lines	Characteristic CPE
HSV	1 to 4	A549	HSV 1: Round, refractile, ballooned cells in foci (uniform cell rounding)
			HSV 2: Round, refractile, ballooned cells in foci (wide variation in cell rounding, including large multi-nucleated cells)
		BGM	Large, web-like foci
		MRC5	Large and small round, refractile cells seen individually or in patches
		RMK^*a*^	Scattered rounded cells with refractile bodies
VZV	5 to 11	A549	“Flattened” appearing cells in foci with small and large rounded cells often with holes in the center of the foci Less round and refractile as HSV
		BGM^*a*^	Compact foci of discohesive cells
		MRC5	Rounded or degenerated cells, usually in an elongated patch
		RMK^*a*^	Circular foci with holes in the center and cytoplasmic bridges between cells
Monkeypox	1 to 4	A549	“Melty” cells in distinct foci, possibly resembling adenovirus
		BGM	Pinching, elongated foci
		MRC5	“Hurricane” pattern, streaming patches of cytopathic effects
		RMK	Refractile cells with pronounced cytoplasmic bridging Rapid degeneration of the cell layer

a CPE may develop more slowly, or not at all, in these cell lines.

## DISCUSSION

Given the relative absence of literature, this study provides pertinent information on the CPE and growth characteristics of MPXV in common cell lines used in a clinical laboratory. We document important morphological characteristics of MPXV, including rapid growth in all 4 cells lines, unique CPE patterns within A549 and MRC-5 cells, and the presence of higher grade CPE in RMK cells. These features may help laboratories that perform viral culture flag specimens as suspicious for MPXV and reduce exposure of lab personnel to high-titer virus.

The available patient characteristics in our cohort are mostly consistent with larger epidemiological trends, showing men between the ages of 20 and 50 being most affected (1). However, several outliers including a 9-year-old and 51-year-old female were also identified. In our cohort, 22% (4/18) of MPXV-positive individuals were women. This is significantly more than the 1.5% of cases reported by the CDC as of August 30^th^, 2022 (19). Perhaps due to a lower pretest probability, the availability of viral culture may have allowed for detection of a pathogen that was not originally included in the provider’s differential diagnosis of female patients. As a reference laboratory, it is difficult to determine which other assays were included with viral culture as part of an individual patients’ work-up. However, when additional infectious disease testing was ordered at ARUP Laboratories, the differential included other viral and bacterial diseases that present with rash or ulcers (e.g., HSV, VZV, *Erhlichia*, and Rickettsia rickettsii).

Descriptions of MPXV CPE have mostly been limited to individual cases or passaged virus (20, 21). Whereas previous studies have shown MPXV growth in VeroE6, MA-104, LLCMK-2, OMK, and BSC-40, our study documents the CPE of direct-from-specimen MPXV in RMK, A549, BGM, and MRC-5 cell lines (16, 17, 20). Detection of MPXV CPE were rarely subtle and in the great majority of cases all cell lines demonstrated either 3+ or 4+ CPE on the day of initial positivity. While CPE were most extensive in RMK cells, all cell lines were capable of CPE formation. There was only 1 specimen (MPXV_0012) in which no CPE were observed in the BGM or MRC5 cell lines on the initial day of positivity (Table S1). This pattern of high grade CPE and broad cell tropism differentiates MPXV from HSV (high grade CPE but frequently limited to A549 and MRC-5 cells) and VZV (CPE slow to develop and restricted to A549 and MRC-5 cells). The elevated CPE of MPXV relative to HSV may reflect differences in viral replication kinetics or viral load; however, it may also be influenced by our limited experience in identifying MPXV from cell culture, potentially missing earlier, more subtle changes.

Evaluation of cell culture for viral CPE can be challenging and some degree of subjectivity is inherent in the method. While not all MPXV culture isolates demonstrated stereotypical CPE, we were able to identify common patterns of CPE and rapid growth kinetics. On occasion, atypical CPE would occur. In several instances, rapid cytolysis of the RMK cells prevented the identification of CPE foci. In one suspected case, rapid 3+ to 4+ CPE appeared in all cell lines but the MRC-5 cell line demonstrated fewer patches of CPE and more individually rounded cells. While suspicious, this was not definitive for MPXV. Subsequent consultation with the ordering provider revealed the patient had a disseminated HSV infection. This diagnosis was confirmed by direct fluorescent antibody testing of the shell vial for HSV.

For 18/19 specimens, the diagnosis of monkeypox virus was confirmed in our laboratory using a orthopoxvirus PCR assay. In the one case which was not tested prior to disposal, the ordering provider confirmed that the specimen tested positive for orthopoxvirus at an outside facility. Compared to specimens that were received for PCR testing only, the Ct values of viral culture samples were significantly earlier (20.8 versus 23.9, *P* < 0.05) highlighting the concern that culture may only detect those samples with very high viral loads. While the presence of culturable virus is not sufficient to prove infectivity, correlation between Ct values and cultivatable virus may provide a useful surrogate marker for infectiousness in epidemiological studies (22). Previous studies have performed culture when Ct values were <32 (17). Within our cohort, the latest Ct observed was 29.0; however, our study was not designed to detect what the Ct limit is for cultivatable MPXV. From a diagnostic standpoint, the low Ct values observed in typical MPX lesions are reassuring that, if future outbreaks were to occur, viral culture would detect a significant number of cases with the benefit of being more robust to viral mutations that may negatively affect molecular methods, as has been observed with TNF receptor gene deletions (23).

Limitations of cell culture compared to molecular techniques are its reduced ability to detect co-infections and biosafety concerns. As viral culture is performed in liquid media, individual colonies cannot be separated as is done with bacteria on solid media. In practice, this means rapidly growing viruses with wide tropism will outcompete slower, more fastidious viruses. This is of particular concern in MPXV infections where longitudinal studies in the Democratic Republic of Congo have found a 12 to 13% coinfection rate of MPXV with VZV in individuals presenting with monkeypox-like illnesses (17, 18). As VZV grows significantly slower than MPXV, these co-infections would likely go undetected by culture. Whether the coinfection rate is similar in the current global outbreak remains to be examined. Finally, performing viral culture places laboratory staff at risk of exposure to high-titer viral specimens. In our lab, employees were offered the JYNNEOS vaccine and specimens were handled using BSL-3 techniques in a BSL-2 environment. Because CPE are insufficient to separate the West African MPXV clade from the Congo Basin MPXV clade, which is considered a select agent under CDC guidelines, shell vials with CPE concerning for monkeypox were immediately destroyed (24). These challenges are less prevalent in molecular testing where specimens can be inactivated prior to handling.

While viral culture is no longer routinely practiced in most clinical microbiology laboratories, the unique advantages it provides help justify its continued existence within large academic medical centers and reference laboratories. Viral culture provides a more hypothesis-free method of detection for rare or emerging viral pathogens, particularly those present at high viral loads in clinical samples. In our laboratory, we detected the first specimen positive for monkeypox virus on July 11th, 2022; 2 weeks prior to the availability of a molecular test in our laboratory. In 7 instances where no orthopoxvirus NAAT was originally ordered, viral culture provided the first clue as to the patient’s diagnosis. Our laboratorians recognized the atypical cell CPE and alerted the medical director who then encouraged the ordering provider to add on orthopoxvirus NAAT. Thus, culture provided important diagnostic information and helped direct patient management.
