# Supplementary material for: Characterization of the Cytopathic Effects of Monkeypox Virus Isolated from Clinical Specimens and Differentiation from Common Viral Exanthems
Source: J Clin Microbiol. 2022 Nov 29;60(12):e01336-22. doi: 10.1128/jcm.01336-22 (PMC9769861; doi:10.1128/jcm.01336-22)
Supplement: Supplemental file 1 — Supplemental material. Download jcm.01336-22-s0001.pdf, PDF file, 6.5 MB [file jcm.01336-22-s0001.pdf]

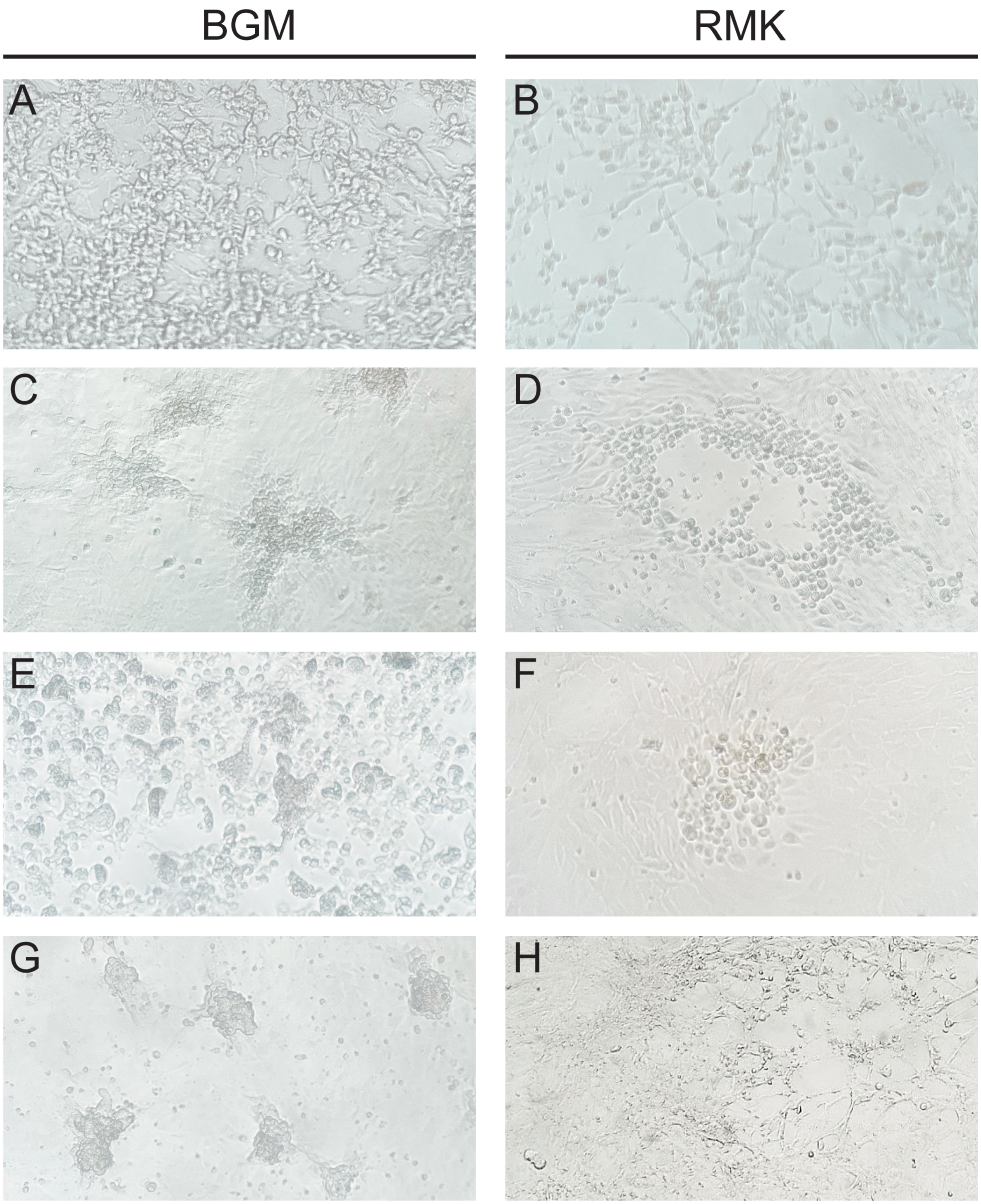

Supplemental Figure 1. Comparison of typical cytopathic effects for MPXV (A, B), HSV1 (C, D), HSV2 (E, F), and VZV (G, H) in BGM and RMK cells. Images taken at 100x magnification.

Supplemental Table 1. CPE intensity on the first day of positive cultures for MPXV, VZV, and HSV1/2 from clinical isolates.

Specimen	First Day CPE Observed	RMK CPE	BGM CPE	A549 CPE	MRC-5 CPE	Ct
MPXV_0001	1	n/a	4+	4+	2+	16.5
MPXV_0002	3	1+	1+	1+	1+	21.3
MPXV_0003	3	4+	4+	4+	4+	23
MPXV_0004	3	4+	4+	4+	4+	21.8
MPXV_0005	4	2+	1+	1+	2+	29
MPXV_0006	2	1+	1+	1+	1+	n/a
MPXV_0007	3	1+	2+	4+	2+	20.3
MPXV_0008	2	4+	3+	4+	3+	24.4
MPXV_0009	2	4+	3+	4+	2+	15.3
MPXV_0010	2	n/a	n/a	2+	1+	23
MPXV_0011	4	4+	3+	1+	4+	23.7
MPXV_0012	4	1+	0	2+	0	20.8
MPXV_0013	2	n/a	n/a	3+	n/a	16
MPXV_0014	2	n/a	n/a	3+	n/a	20
MPXV_0015	3	4+	4+	3+	4+	20.3
MPXV_0016	4	3+	2+	1+	1+	24.2
MPXV_0017	2	3+	3+	4+	4+	16.7
MPXV_0018	2	n/a	n/a	4+	n/a	18.2
MPXV_0019	2	4+	4+	4+	4+	19.5
VZV_0001	10	0	1+	1+	0	
VZV_0002	6	0	0	0	1+	
VZV_0003	5	0	0	0	1+	
VZV_0004	11	1+	0	1+	0	
VZV_0005	7	1+	1+	1+	1+	
VZV_0006	9	0	0	1+	1+	
VZV_0007	11	0	0	1+	0	
VZV_0008	9	0	0	1+	0	
VZV_0009	11	0	0	0	1+	
VZV_0010	7	0	1+	1+	0	
VZV_0011	9	0	1+	1+	0	
HSV_0001	1	0	0	1+	0	
HSV_0002	1	0	0	1+	0	
HSV_0003	2	0	1+	1+	1+	
HSV_0004	1	1+	1+	0	1+	
HSV_0005	1	4+	4+	4+	4+	
HSV_0006	1	0	1+	2+	1+	
HSV_0007	2	3+	4+	4+	4+	

Private Information

HSV_0008	7	0	2+	2+	0	
HSV_0009	1	0	1+	2+	1+	
HSV_0010	3	0	0	2+	0	
HSV_0011	2	0	2+	3+	3+	
HSV_0012	2	1+	4+	4+	4+	
HSV_0013	2	1+	3+	4+	4+	
HSV_0014	3	0	2+	2+	3+	
HSV_0015	1	0	1+	2+	1+	
HSV_0016	2	0	1+	1+	0	
HSV_0017	2	1+	3+	4+	4+	
HSV_0018	1	1+	1+	1+	1+	
HSV_0019	1	0	1+	4+	3+	
HSV_0020	3	4+	4+	4+	4+	

CPE graded as follows: 0 = no CPE present, 1+ = ≤25% CPE, 2+ = >25% CPE, 3+ = >50% CPE, 4+ = >75% CPE.

Private Information
